# Seasonal forecasts as public health tools: Preparing Latin America for the 2026 El Niño

**DOI:** 10.1371/journal.pgph.0006570

**Published:** 2026-07-08

**Authors:** Yasna Palmeiro-Silva, Jeremy J. Hess, Kristie L. Ebi

**Affiliations:** Center for Health and the Global Environment, Department of Global Health, University of Washington, Seattle, Washington, United States of America; PLOS: Public Library of Science, UNITED STATES OF AMERICA

## Seasonal forecasts as public health tools: preparing Latin America for the 2026 El Niño

The tropical Pacific is signalling the development of a likely moderate (with possibilities of strong) El Niño event by mid-2026. As of June 2026, the C3S multi-system shows most Niño 3.4 sea-surface temperature forecasted relative anomalies (vs 1991–2020 period) falling between 2.3 °C and 3.9 °C by December 2026, with more than 50% of the models exceeding 3 °C amplitude [1] (the 2015/16 anomaly reached 3 °C [2]). NOAA’s Climate Prediction Center indicates El Niño is likely to emerge (82% chance) in May-July, lasting until the end of 2026 and beginning of 2027 [3], whilst the IRI/Columbia plume forecasts sustain probabilities of 72–80% [4]. Although these early forecasts are still associated with large uncertainties, there is a strong consensus of a warming signal and that El Niño conditions are likely to develop in May-July and maintained over 2026–27.

While there is uncertainty in the forecast, and this uncertainty may be amplified by the added challenge of climate change, action to protect health is warranted based on the substantial evidence of adverse health impacts associated with prior El Niño events in the region [5,6]. Forecasting challenges notwithstanding, under the Precautionary Principle, there is little downside to investments in enhanced preparedness, and the upside may be considerable.

Latin America and the Caribbean (LAC) is the second most disaster-prone region globally, with over 1,500 disasters affecting more than 190 million people since 2000 [7]. The teleconnections of El Niño across the region are well characterised: hotter weather overall, heavy rainfall in northern Mexico, Peru, Ecuador, and parts of South America (Chile and Uruguay), and droughts in Central America, northern Brazil, and Colombia and Venezuela [8]. These extremes cascade into ecosystems and human systems, increasing the risk of heatwaves, wildfires, flooding, landslides, reduced crop yields, and diverse health outcomes through multiple pathways.

The estimated life expectancy losses following the 1997–98 El Niño events across Pacific Rim countries were of 0.4 years [9]. During the 2015–16 El Niño, flooding resulted in more than 150,000 people displaced and 100 people dead, in addition to worsened food insecurity and health alerts for mosquito-borne diseases [10]. The 2023–24 El Niño contributed to prolonged droughts in Central America’s Dry Corridor and northern South America, while driving intense rainfall and flooding in Ecuador, Chile, and Peru, disrupting agricultural production and increasing food insecurity. Concurrently, the region recorded its worst dengue season on record, with reported cases increasing by 600% across the Andean and Southern Cone countries (Brazil and Paraguay heavily affected) [11]. During 2023, coinciding with El Niño onset, LAC recorded an estimated 8,436 heatwave-related excess deaths [12].

Unfortunately, these numbers are likely to be conservative given the scarcity of systematic data collection and analysis associated with El Niño-related health disasters in Latin America. Additionally, if the 2026 event reaches the intensities currently forecast, these health impacts are likely to be amplified considerably in a warmer planet, particularly affecting communities contending with social inequities, fragile health systems, urban informality, food insecurity, lingering effects of other social and health challenges, including the effects of global conflicts.

**Fig 1:**
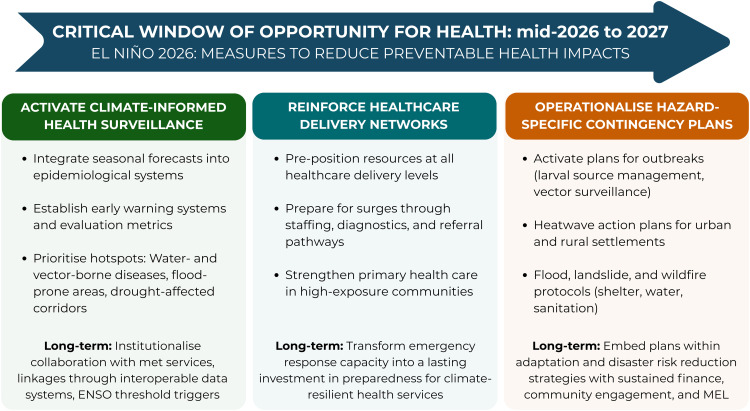
Actions and measures to reduce preventable health impacts amidst 2026 El Niño event. ENSO: El Niño-Southern Oscillation; MEL: Monitoring, Evaluation, and Learning. Source: Author’s creation.

Taking advantage of the progress in seasonal forecasting, the months ahead represent a critical public health opportunity and window for anticipatory action to reduce El Niño-related preventable health impacts in Latin America via three main actions (Fig 1):

i **Climate-informed health surveillance must be activated now, not when impacts arrive.** In the short-term, the forecasting of El Niño offers a critical window for anticipatory action and early warning; therefore, public health systems across Latin America should integrate seasonal forecasts into epidemiological surveillance [13], prioritising water- and vector-borne disease hotspots, flood-prone settlements, and drought-affected corridors. Over the longer term, governments should institutionalise these linkages through permanent platforms, interoperable data systems between ministries of health and meteorological services, and binding protocols that trigger pre-agreed public health responses when El Niño-Southern Oscillation (ENSO) thresholds are forecast.
ii **Resources at different levels of healthcare delivery (e.g., primary, secondary, tertiary) must be pre-positioned and primary health care strengthened in high-exposure communities.** Historical evidence consistently demonstrates that the most significant health impacts of El Niño are observed in the year following onset [14]. This means the window from mid-2026–2027 is a critical period for continuing action. Primary health care networks must be reinforced to manage anticipated surges in respiratory illness, diarrhoeal disease, vector-borne diseases, and heat-related morbidity, with targeted staffing, diagnostic capacity, and referral pathways. Beyond this event, Latin American governments should translate emergency surge capacity into durable investment in climate-resilient primary care, treating ENSO as a recurring stress test for health system preparedness rather than as isolated emergencies.
iii **Hazard-specific contingency plans, including risk communication, must be operationalised soon.** In the coming months, national and subnational authorities should activate anticipatory response plans covering water- and vector-borne disease outbreaks (including intensified larval source management, vector surveillance, and timely deployment of diagnostic and clinical guidelines); food security responses; heatwave action plans for urban and rural settlements; and flood and landslide protocols linking shelter, water-and-sanitation, and disease surveillance sectors. In the longer term, these contingency plans should be embedded within national adaptation and disaster risk reduction strategies, with sustained financing, community co-design in Indigenous and informal-settlement contexts, and periodic evaluation after each ENSO cycle to ensure they evolve with a warming climate [5].

In sum, advances in seasonal forecasting now afford Latin America a tangible lead time that should be harnessed for public health preparedness, particularly as a warming planet amplifies El Niño-related hazards. This event poses a challenge and test to public health systems and health care delivery in Latin America, a region increasingly attentive to climate change health impacts. Prompt action can transform the challenge into an opportunity for the region to demonstrate its readiness and commitment to population health.

## References

[1] The Copernicus Programme. Seasonal forecasts [Internet]. Copernicus EU; 2026 [cited 2026 June 5]. Highlights of the latest seasonal forecasts. Available from: https://charts.ecmwf.int/products/seasonal_system5_nino_plumes?base_time=202606010000&nino_area=NINO3-4_rel

[2] Stockdale T, Balmaseda, Ferranti L. The 2015/2016 El Niño and beyond [Text] [Internet]. ECMWF; 2017 [cited 2026 Apr 10]. Available from: https://www.ecmwf.int/en/newsletter/151/meteorology/2015-2016-el-nino-and-beyond

[3] NOAA Climate Prediction Center/NCEP/NWS. Climate Prediction Center: ENSO Diagnostic Discussion [Internet]. NOAA; 2026 [cited 2026 May 15]. Available from: https://www.cpc.ncep.noaa.gov/products/analysis_monitoring/enso_advisory/ensodisc.shtml

[4] IRI – International Research Institute for Climate and Society. ENSO Forecast [Internet]. 2026 [cited 2026 Apr 10]. Available from: https://iri.columbia.edu/our-expertise/climate/forecasts/enso/current/

[5] Rony MKK, Wahiduzzaman M, Rahman MM, Bala SD. Impact of El Niño on public health and its preparedness measures. Bull Natl Res Cent. 2024;48(1):4. doi: 10.1186/s42269-023-01160-4

[6] Li G, Lu P, Weinstein P, Urban A, Tong S, Ryti N, et al. The burden of El Niño-Southern Oscillation-related dengue attributable to anthropogenic climate change: a multicountry modelling study. Lancet Planet Health. 2026;10(4):101454. doi: 10.1016/j.lanplh.2026.101454 42044647

[7] OCHA, UNDRR. Overview of disasters in Latin America and the Caribbean 2000-2022. OCHA; 2023.

[8] Ropelewski CF, Halpert MS. Global and Regional Scale Precipitation Patterns Associated with the El Niño/Southern Oscillation. Mon Wea Rev. 1987;115(8):1606–26. doi: 10.1175/1520-0493(1987)115<1606:garspp>2.0.co;2

[9] Xu Y, Zhu W, Samanta D, Horton BP. Enduring impacts of El Niño on life expectancy in past and future climates. Nat Clim Chang. 2026;16(2):148–54. doi: 10.1038/s41558-025-02534-4

[10] CIIFEN. El Niño 2015-16: evolución, vulnerabilidad e impactos en Latinoamérica. 2017.

[11] Ortiz-Prado E, Izquierdo-Condoy JS, Vásconez-González J. Urgent Response Needed: Addressing the Dengue Crisis in the Andean and Southern Cone Latin American Regions. Am J Trop Med Hyg. 2024;111(4):714–8. doi: 10.4269/ajtmh.24-0178 39106852 PMC11448526

[12] Hundessa S, Huang W, Xu R, Yang Z, Zhao Q, Gasparrini A, et al. Global excess deaths associated with heatwaves in 2023 and the contribution of human-induced climate change. Innovation (Camb). 2025;6(10):101110. doi: 10.1016/j.xinn.2025.101110 41084606 PMC7618246

[13] Romanello M, Walawender M, Hsu SC, Moskeland A, Palmeiro-Silva Y, Scamman D, et al. The 2025 report of the Lancet Countdown on health and climate change: climate change action offers a lifeline. The Lancet. 2025;406(10521):2804–57. doi: 10.1016/S0140-6736(25)01919-1 41175887

[14] World Health Organization. Public Health Situation Analysis. El Niño Global Climate Event [Internet]. Geneva, Switzerland: WHO; 2023. Available from: https://cdn.who.int/media/docs/default-source/2021-dha-docs/phsa-el-nino-2023_final_na.pdf?sfvrsn=53

